# Effect of neuroticism on Chinese athletes’ vigor: serial mediating roles of pre-competition anxiety and mind wandering

**DOI:** 10.3389/fpubh.2024.1412203

**Published:** 2024-11-22

**Authors:** Jieling Li, Chuangye Li, Bao Tian

**Affiliations:** ^1^School of Physical Education, Hebei Normal University, Shijiazhuang, China; ^2^Key Laboratory of Measurement and Evaluation in Exercise Bioinformation of Hebei Province, Shijiazhuang, China; ^3^Physical Education Postdoctoral Research Station, Hebei Normal University, Shijiazhuang, China; ^4^School of Psychology, Capital Normal University, Beijing, China

**Keywords:** neuroticism, athlete, mind wandering, vigor, anxiety

## Abstract

**Introduction:**

Vigor plays an important role in mental health, and it is closely related to sporting performance. Neuroticism can affect individual vigor, but its internal mechanism remains unclear. This study aims to examine the relationship between neuroticism and vigor, and the role of anxiety and mind wandering between the two.

**Methods:**

A total of 591 athletes completed questionnaires on neuroticism, pre-competition anxiety, mind wandering and vigor. The survey data were tested for common method biases, Pearson’s correlation, and structural equation model via SPSS 25.0 and Mplus 7.0.

**Results:**

Results showed that neuroticism had a predictive effect on athletes’ vigor 4 (*β* = −0.511, *p* < 0.001). Pre-competition anxiety (*β* = −0.056, BC 95% CI = [−0.091, −0.028]) and mind wandering (*β* = −0.030, BC 95% CI = [−0.054, −0.014]) mediate neuroticism effects on vigor separately. There was a significant serial mediation effect from “neuroticism→Pre-competition anxiety→mind wandering→energy” (*β* = −0.010, BC 95% CI = [−0.023, −0.002]).

**Discussion:**

This study provides a reference for the systematic investigation of the relationship between neuroticism and vigor, and specific intervention methods for ensuring athletes’ vigor and improving sports performance.

## Introduction

With researchers calling for the study of positive mental abilities, vigor is getting a lot of attention in many fields (1–3). Vigor broadly refers to a positive affect denoting a combination of contentment and a positive energy balance (4). In specific studies, researchers have defined vigor in many ways, such as a multidimensional construct representing individuals’ feelings (5, 6), positive affective arousal (7), energetic arousal (8) or the positive characteristic of the profile of mood states (9). It’s worth noting that individuals with high level of vigor are not easily exhausted and tend to persevere in the face of major social challenges (10). In addition, the positive impact of vigor contributes to key work processes and influence key organizational outcomes, including performance (11). In sports, the professional lives of many athletes have evolved into job-like careers (12). Therefore, sport is their ‘job’ for (semi-) professional athletes. Hence, similar processes occurring in the workplace may take place in the domain of sport as well (13). As with other workplaces, studies have shown that vigor is positively correlated with athletic performance (14). According to a study of 576 Brazilian elite athletes 60 min before the start of a competition, athletes who perform better in sports are characterized by high level of vigor in the profile of mood states (15).

Coaches and researchers have been searching for factors that influence vigor to improve athletes’ vigor levels, and neuroticism is one of the main factors. Individuals with a high level of neuroticism are easily affected by negative emotions and experiencing difficulties to be patient, calm, and confident when facing problems. They face problems in communicating with their peers and are prone to conflicts (16). Since vigor contains multidimensional structure, many studies focused on work engagement (17, 18). Work engagement is characterized by a positive motivational state of dedication, that is, strong involvement in one’s work and a feeling of being energetic (19). Janssens and colleagues (20) found that a higher level of neuroticism is correlated with a lower level of vigor and dedication in Flemish workers. Martos Martínez and colleagues (21) found that neuroticism has a negative relationship with work engagement in nursing professionals. Therefore, neuroticism may affect vigor levels. In addition, neuroticism levels correlate with competitive performance. Individual sports champions were characterized by a lower level of neuroticism (22). So, whether neuroticism has a similarly negative effect on an individual’s vigor in the field of sport was a question worth exploring.

However, the mechanisms of how neuroticism affects an individual’s vigor is unclear. Anxiety is a compound emotion consisting of fear, guilt, pain, and anger (23), which is a negative emotional experience that often arises in competition and training. It is often perceived as the athlete’s “public enemy” and can prevent the athlete from performing at his or her normal level (24). Anxiety differs from neuroticism in that anxiety is a type of negative emotion, while neuroticism belongs to personality traits. However, neuroticism is the personality trait most closely associated with emotions, such as anxiety. Research has shown that college students with high neuroticism is prone to feelings of anxiety (25). In addition, anxiety is closely related to depressive symptoms in Chinese collegiate athletes (26), and the production of negative emotions can affect the positive emotions related to individual’ vigor. Athletes may also lead to anxiety when faced with the expectation of achieving excellence that is placed on them by their country and the audience. The above psycho-social factors that lower one’s ability to have positive feelings (27). Accordingly, it can be assumed that anxiety may play a mediating role between neuroticism and vigor.

In addition, anxiety is closely related to mind wandering. Some researchers define mind wandering as a situation in which executive control shifts away from a primary task to the processing of personal goals, individuals lack control in this process (28). In fact, mind wandering is not only related to personal goals, but also to other recent events (29). Mind wandering is different from distraction. When an individual undergoes mind wandering, attention is diverted to internal mental processes; however, during distraction, attention is diverted to other stimuli in the external environment (30). Mind wandering, which is endogenous to an individual’s thought, is a frequent phenomenon in human beings, and it accounts for 30–50% of people’s waking time (31). An experimental study of psychology undergraduates found that anxiety was positively associated with the frequency of mind wandering (32). According to the “Attentional Control Theory” of anxiety (33), anxiety impairs individuals’ attentional control by interfering primarily with inhibitory and switching functions, thus reducing processing efficiency. The failure of control model of mind wandering proposed that a failure of attentional control leads to increased tendency of mind wandering (34, 35). Also, mind wandering leads to changes in an individual’s mood state. A considerable number of research were conducted on mind wandering in relation to emotions, the results suggest that mind wandering is accompanied by negative emotional experiences for individuals (36–38). So, mind wandering may affect individual’ vigor levels to some extent. Furthermore, it has also been shown that individuals with neurotic personalities are more prone to mind wandering (35, 39). These above findings suggest anxiety and mind wandering may play serial mediating role between neuroticism and vigor. The IPACE (Interaction of Person-Affect-Cognition-Execution) model provides an indirect theoretical basis for the relationship between the above four variables. The I-PACE model summarized the mechanisms underlying the development and maintenance of specific Internet-use disorders (40). Applying the I-PACE model to athlete, the “person” refers to the personality in this model corresponding to “neuroticism,” the “affect” in this model corresponding to “pre-competition anxiety,” the “cognition” in this model corresponding to “mind wandering” and the “execution” in this model corresponding to “vigor.” At the same time, a study has shown that neuroticism indirectly influence mind wandering through trait anxiety (41). However, there is a lack of exploring the mechanisms among the four factors. Therefore, the present study focuses on the pathways through which neuroticism affects vigor and whether it affects vigor indirectly through anxiety and mind wandering.

**Figure 1:**
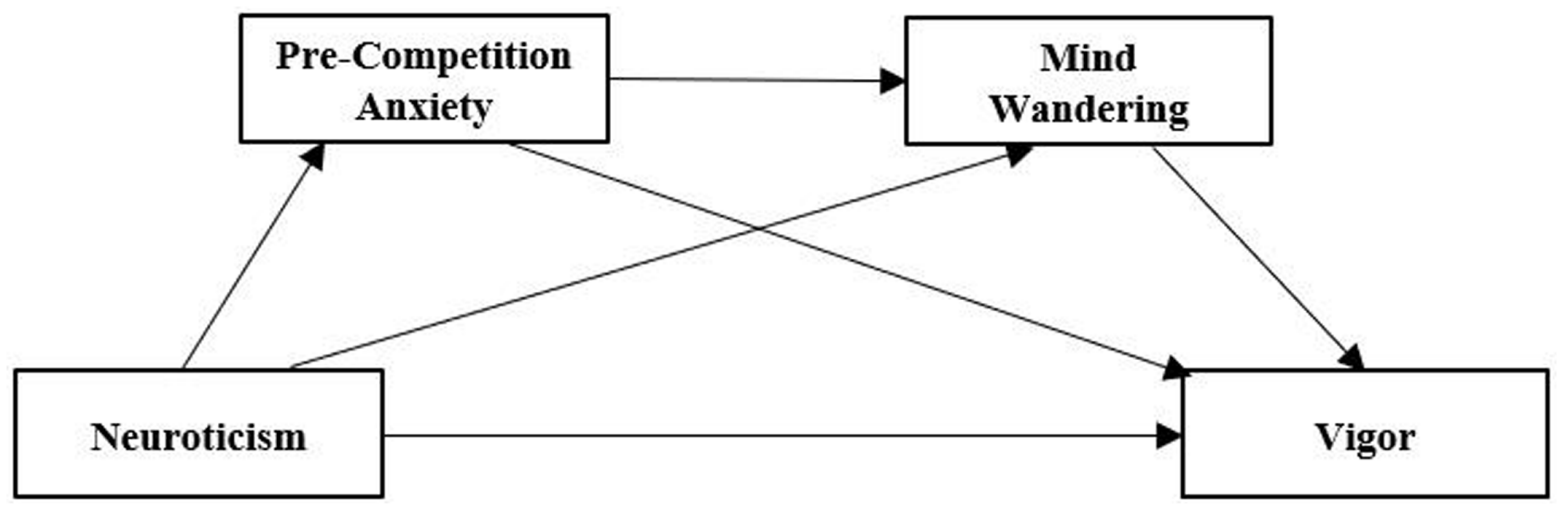
The serial mediation model hypothesis of pre-competition anxiety and mind wandering between neuroticism and vigor.

In summary, this study not only attempts to test the effect of neuroticism on athletes’ vigor but also explains the underlying mechanisms. Based on previous studies on neuroticism, vigor, anxiety and mind wandering, the following hypotheses are proposed in the field of sports (Figure 1).

*H1*: Neuroticism has a negative effect on athletes’ vigor.

*H2*: Pre-competition anxiety plays a mediating role between neuroticism and vigor of athletes.

*H3*: Mind wandering mediates between neuroticism and athlete’ vigor.

*H4*: Athlete anxiety is positively related to mind wandering.

*H5*: Neuroticism has a negative effect on vigor through the serial mediation of pre-competition anxiety and mind wandering.

## Methods

### Participants

Participants were obtained by convenience sampling and were located in Hebei, Henan, Zhejiang, Sichuan, Tianjin, Shanghai, and Beijing in China. All participants must have trained at least 1 year and have participated in the provincial level competitive events. Questionnaires were distributed in the form of online^1^ and paper versions. Data were collected in March 2023. A total of 727 responses were recovered, and after those with incorrect answers on three forced-choice questions and irregular answers were excluded, a final valid scale of 591 responses was obtained. The recovery rate of valid responses was 81.29%. The final sample included 303 males and 288 females. The average age of the participants was 19.323 years (SD = 4.082). They had participated with their respective teams for an average of 6.382 years (SD = 3.656).

The study was reviewed by the Ethics Committee of Hebei Normal University (2023LLSC031), and all subjects who participated in the survey provided informed consent.

### Measures

#### Chinese adjective scale of big five personality brief version

The Chinese Adjective Scale of Big Five Personality Brief version was used to conduct the 4 items of the neuroticism dimension of athletes’ personality, using a six-point scale (42). 1 item was deleted by correcting the index to meet the standard of model fit, leaving three items. The internal consistency reliability of the neuroticism dimension was 0.784 (42).

#### Profile of mood states

The vigor dimension of this scale was selected, with 6 items, using a five-point scale (43). 1 item was deleted by correcting the index to meet the criteria for model fit, leaving five items. The reliability of the vigor dimension with Chinese respondents was 0.71 (43).

#### Athletes’ mind wandering scale

The frequency of mind wandering in athletes was measured by the Athletes’ Mind Wandering Scale, 3 dimensions: psychological gap, competition mood, and somatic sensation (44). The scale has a total of 12 items and a five-point scale. It can investigate the situations in which athletes are more likely to have mind wandering in training and competition, and the total score represents the frequency of mind wandering in athletes. The internal consistency reliability for each dimension were 0.710, 0.800 and 0.826; the scale had good construct validity (*χ*^2^*/df* = 2.968, TLI = 0.911, AGFI = 0.894, CFI = 0.924, RMSEA = 0.059) (44).

#### Pre-competition emotion scale-T brief version

The Pre-Competition Emotion Scale-T Brief version was used to measure athletes’ pre-competition anxiety (45). It consists of four dimensions, namely, individual failure anxiety, self-confidence, social expectancy anxiety, and somatic anxiety, with a total of 16 items and a six-point scale. The internal consistency reliability for each dimension were 0.86, 0.85, 0.85 and 0.82; The scale had good construct validity (*χ*^2^*/df* = 2.42, NNFI = 0.91, AGFI = 0.90, CFI = 0.92, RMSEA = 0.05) (45).

### Data analysis

Correlation analysis of the measured demographics and related scale scores of the study participants was conducted using SPSS 22.0. Validation factor analysis was conducted using Mplus 7.0 was used to test the reliability, convergent validity, model fit, and serial mediation effects. The bootstrap method of mediation analysis was used, which is the process of drawing a large bootstrap sample (sample size = 1,000 in this study) and obtaining statistics through repeated sampling with put-backs. This method does not require normality assumptions nor large samples, and it is useful for analyzing small-to-medium samples. The commonly used bootstrap methods include the percentile bootstrap method and the bias-corrected percentile bootstrap method, both of which are presented in the results section of this study. A significant indirect effect is indicated when the confidence interval does not contain 0.

## Results

### Common method bias test

This study used the Harman one-way method to conduct the common method bias test of the factors to verify whether an obvious systematic error exists in the survey. The results showed that out of 51 factors, eight had eigenvectors greater than 1, and the first factor explainable percentage was 27.67%, which was lower than 40%. Therefore, the factors used in this study had no significant common method bias.

### Multicollinearity test

In order to test whether there is a problem of multicollinearity due to high correlation between the independent variables, we conducted a test by variance inflation factor (VIF). The results showed that the VIF values of the independent variables involved in this study are 1.222, 1.326 and 1.532, which are less than 10. Therefore, there is no multicollinearity among the independent variables.

### Reliability and validity tests of the scale

As shown in Table 1, the composite reliability (CR) and convergent validity were calculated to test the reliability of the items and the ability of the dimensions to explain the items. The CR of the dimensions all above 0.7. Convergent validity is assessed by average variance extracted (AVE), which is the average ability of the dimensions to explain the items, was greater than 0.36 for all dimensions, indicating that it was in the acceptable range. The correlation coefficients between the dimensions and those between the dimensions and the sub-scales to which they belonged were calculated for comparison to test the discriminant validity between the dimensions, as shown in Table 1. The correlation coefficients between each dimension and its affiliated sub-scales were basically higher than those with other dimensions, indicating that the discriminant validity of each dimension was good.

**Table 1:** Reliability and validity.

Dim.	NEU	VIGOR	PG	CM	SS	IFA	SEA	SA	SC
NEU	**0.758**								
VIGOR	−0.437	**0.794**							
PG	0.161	−0.228	**0.729**						
CM	0.201	−0.226	0.588	**0.713**					
SS	0.147	−0.200	0.619	0.559	**0.721**				
IFA	0.303	−0.301	0.290	0.407	0.306	**0.782**			
SEA	0.280	−0.252	0.302	0.338	0.368	0.686	**0.754**		
SA	0.301	−0.286	0.300	0.352	0.369	0.611	0.661	**0.737**	
SC	0.378	−0.453	0.187	0.303	0.146	0.350	0.225	0.244	**0.743**
CR	0.802	0.894	0.773	0.805	0.843	0.862	0.840	0.826	0.829
AVE	0.575	0.630	0.532	0.509	0.520	0.612	0.569	0.543	0.552

The diagonal bold is the Pearson correlation between dimensions and the sub-scale to which they belong, and the lower triangle is the Pearson correlation between dimensions. NEU, neuroticism; PG, psychological gap; CM, competition mood SS: somatic sensation; IFA, individual failure anxiety; SEA, social expectancy anxiety; SA, somatic anxiety; SC, self-confidence; CR, composite reliability; AVE, average variance extracted.

### Correlation analysis among neuroticism, mind wandering, pre-competition anxiety, and vigor

The correlation analysis showed that the athletes’ neuroticism had a significant negative correlation with vigor and a significant positive correlation with mind wandering and pre-competition anxiety. A significant negative correlation was found between vigor and mind wandering and pre-competition anxiety. Moreover, mind wandering and pre-competition anxiety had a significant positive correlation, as shown in Table 2. The athletes’ years of training and age had a significant positive correlation with the frequency of mind wandering. Therefore, the athletes’ years of training and age were analyzed as control variables in the following analyses.

**Table 2:** Descriptive statistics and correlation analysis results of neuroticism, mind wandering, pre-competition anxiety and vigor.

Variable	M ± SD	1	2	3	4	5	6	7
1. NEU	11.692 ± 4.350	1						
2. VIGOR	19.621 ± 4.708	−0.437**	1					
3. MW	24.787 ± 7.904	0.200**	−0.255**	1				
4. PCA	47.740 ± 14.787	0.402**	−0.409**	0.467**	1			
5. LEVEL	3.208 ± 0.886	−0.019	0.010	−0.010	0.015	1		
6. YT	6.382 ± 3.656	0.005	0.021	0.094*	−0.035	−0.419**	1	
7. AGE	19.323 ± 4.082	−0.007	0.041	0.249**	−0.014	−0.069	0.507**	1

*p < 0.05; **p < 0.01; MW, mind wandering; PCA, pre-competition anxiety; LEVEL, skill level of athlete; YT, years of training.

### Analysis of direct and indirect effects of neuroticism on vigor

On the basis of correlation analysis, the direct and indirect effects of neuroticism on vigor were further examined. Among them, the recommended value of model fit, χ^2^*/df*, is as small as possible, which is within 5 according to the wider standard identified by Schumacker and Lomax (46). With regard to other indicators, RMSE is generally recognized to be less than 0.08, SRMR is less than 0.08, CFI is more than 0.9, and TLI should be more than 0.9. A second-order modeling analysis is required to test whether factors in the first-order are measuring factors in a higher order. The target coefficient (TC) is generally used as a criterion to judge whether the second-order CFA could replace the first-order CFA. The formula is as follows: TC = first-order CFA fully correlated chi-square value/s-order CFA cardinality. The closer the target coefficient is to 1, the more the second-order model could appropriately represent the first-order model, and reaching 0.74 is generally considered to be acceptable (47). In the present study, mind wandering and pre-competition anxiety were second-order measurement models, and the TC of the pre-competition anxiety model = 429.470/450.234 = 0.954, which met the criterion of 0.74. A notable detail that mind wandering had three first-order models only, and the method of determination is not the same as that in pre-competition anxiety model. When determining whether the hypothesized model could be further refined from a first-order model to a second-order model, if the second-order CFA has only three first-order factors, it is referred to as an equivalent model, indicating that the second-order CFA and the first-order fully correlated CFA have the exact same model fit. At this time, the judgment criterion is the factor load value which greater than 0.7 and more than 0.6 are acceptable, and it indicates that second-order CFA can replace the first-order CFA model (47). The factor loading of second-order to first-order factors in the present study were 0.935, 0.804, and 0.842, indicating that the second-order CFA model of the Athletes’ Mind Wandering Scale can replace the first-order CFA model.

First, the direct effect of neuroticism on vigor was examined by constructing a structural equation. The model fit was good, and the fit indices were χ^2^*/df* = 1.914, RMSEA = 0.039, SRMR = 0.022, CFI = 0.990, and TLI = 0.986. The results showed that the direct effect of neuroticism on vigor was significant (*β* = −0.511, *p* < 0.001). The results support hypothesis 1.

**Figure 2:**
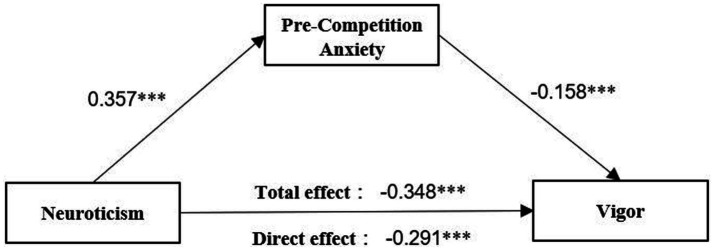
The mediating pathway of pre-competition anxiety affecting vigor.

Second, a mediation model was constructed with neuroticism as the independent variable, pre-competition anxiety as the mediator variable, and vigor as the dependent variable. The model fit indices were good, with fit indices of χ^2^*/df* = 3.127, RMSEA = 0.060, SRMR = 0.073, CFI = 0.925, and TLI = 0.916. All fit statistics were within reasonable ranges, and the results of the analysis support the rationality of the model initially constructed in this study. As shown in Figure 2, the model path demonstrated a significant positive effect of neuroticism on pre-competition anxiety (*β* = 0.357, *p* < 0.001) and a significant negative effect of pre-competition anxiety on vigor (*β* = −0.158, *p* < 0.001). Meanwhile, had a significant negative direct effect on vigor (*β* = −0.291, *p* < 0.001). The results also showed a significant indirect effect of “neuroticism→pre-competition anxiety→vigor” (*β* = −0.056, BC 95% CI = [−0.091, −0.028], percentile 95% CI = [−0.090, −0.027]), with confidence intervals that did not include 0.

**Figure 3:**
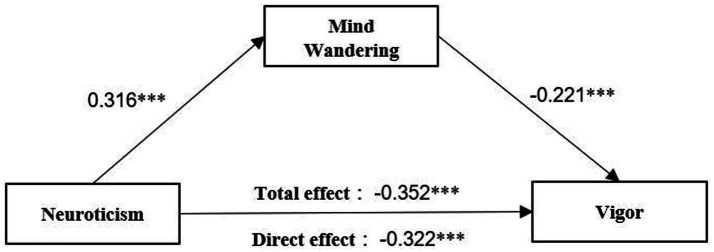
The mediating pathway of mind wandering affecting vigor.

Third, a mediation model was constructed with neuroticism as the independent variable, mind wandering as the mediator variable, and vigor as the dependent variable. The model fit indices were good, with fit indices of χ^2^*/df* = 2.562, RMSEA = 0.051, SRMR = 0.044, CFI = 0.939, and TLI = 0.931. All fit statistics are within a reasonable range, and the analysis results support the rationality of the model initially constructed in this study. As shown in Figure 3, the model path exhibited a significant positive effect of neuroticism on mind wandering (*β* = 0.136, *p* < 0.001) and a significant negative effect of mind wandering on vigor (*β* = −0.221, *p* < 0.001). Meanwhile, a significant negative direct effect of neuroticism on vigor (*β* = −0.322, *p* < 0.001) was found. The results also showed a significant indirect effect of “mind wandering→vigor” (*β* = −0.030, BC 95% CI = [−0.054, −0.014], percentile 95% CI = [−0.051, −0.012]), with confidence intervals that did not contain 0.

**Figure 4:**
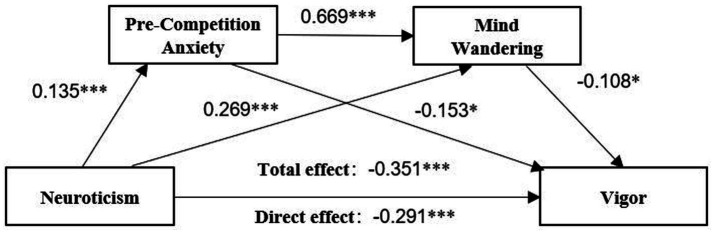
The serial mediating pathway of pre-competition anxiety and mind wandering affecting vigor.

Finally, the serial mediator effect was verified with neuroticism as the independent variable, pre-competition anxiety and mind wandering as the mediator variables, and vigor as the dependent variable. The model fit indices were good, and the fit indices were χ^2^*/df* = 2.459, RMSEA = 0.050, SRMR = 0.063, CFI = 0.910, and TLI = 0.903. All fit statistics are within a reasonable range, and the analysis results support the rationality of the model initially constructed in this study. As shown in Figure 4, the model path demonstrated that neuroticism had a positive effect on pre-competition anxiety (*β* = 0.135, *p* < 0.001), and pre-competition anxiety had a positive effect on mind wandering (*β* = 0.669, *p* < 0.001); mind wandering has a negative effect on vigor, (*β* = −0.108, *p* < 0.05). Neuroticism exhibited a negative direct effect on energy, (*β* = −0.291, *p* < 0.001) and a positive effect on mind wandering (*β* = 0.269, *p* < 0.001), and pre-competition anxiety had a negative effect on vigor (*β* = −0.153, *p* < 0.05). All the results of mediating effects are shown in Figure 4 and Table 3. Specifically, the serial mediating effects of mind wandering and pre-competition anxiety are composed of indirect effects produced by the following three paths: (1) an indirect effect generated by neuroticism→pre-competition anxiety→vigor (*β* = −0.029, BC 95% CI = [−0.058, −0.005], percentile 95% CI = [−0.057, −0.004]); (2) an indirect effect generated by neuroticism→mind wandering→vigor (*β* = −0.021, BC 95% CI = [−0.043, −0.001], percentile 95% CI = [−0.043, −0.001]); (3) an indirect effect produced by neuroticism→pre-competition anxiety→mind wandering→vigor (*β* = −0.010, BC 95% CI = [−0.023,-0.002], percentile 95% CI = [−0.022, −0.001]). The confidence intervals that did not contain 0 indicated that all of the above indirect effects were significant.

**Table 3:** The serial mediating effect of pre-competition anxiety and mind wandering on neuroticism and vigor.

		Product of Coefficient			BOOTSTRAP 1000 TIMES 95% CI			
Mediation path	Point				Bias corrected		Percentile	
	Estimate	S.E.	Est./S.E.	*p*-value	Lower	Upper	Lower	Upper
NEU → MW → VIGOR	−0.021	0.010	−1.983	0.047	−0.043	−0.001	−0.043	−0.001
NEU → PCA → VIGOR	−0.029	0.014	−2.112	0.035	−0.058	−0.005	−0.057	−0.004
NEU → PCA → MW → VIGOR	−0.010	0.005	−1.889	0.059	−0.043	−0.001	−0.043	−0.001

*p < 0.05; **p < 0.01; NEU, neuroticism; MW, mind wandering; PCA, pre-competition anxiety.

## Discussion

The relationship between neuroticism and vigor and the role of anxiety and mind wandering between them were explored by constructing structural equations. The results showed that neuroticism has a negatively predictive effect on athletes’ vigor, pre-competition anxiety and mind wandering act as serial mediators.

First, the direct effect of neuroticism on vigor was examined. The results indicated that the direct effect of neuroticism on vigor was significant, and neuroticism negatively predicted vigor. These findings support hypothesis 1. The reasons for this result should go back to the specific characteristics of neuroticism. For example, a study suggest that individuals with high neuroticism show stronger emotional reactions and poorer emotional perception and coping (48). Therefore, individuals with neuroticism can experience more negative emotions, while vigor belongs to positive emotions. Thus, having more negative emotions affects the positive emotions of individuals, which could then easily affect their vigor level and harm their performance. This result indicates that neurotic personality is an important factor affecting athletes’ vigor. Specifically, athletes with high neuroticism are more likely to show a lower vigor state.

This study found that neuroticism had a significant positive effect on pre-competition anxiety. This is similar to the findings of previous studies that neurotic personality is significantly associated with a greater level of anxiety (25, 49). Meanwhile, pre-competition anxiety had a significant negative effect on vigor, a significant indirect effect of “neuroticism→pre-competition anxiety→vigor” was found, indicating that pre-competition anxiety mediated the relationship between neuroticism and vigor. This finding is consistent with hypothesis 2. Athletes are often under pressure prior to competition, expectations from coaches and family members, or the expectation to win honors for the country, which can cause pre-competition anxiety to elevate. Pre-competiton anxiety consists of four dimensions (see Table 1 for the results of the correlation analysis). IFA, SEA and SA refer to individual failure anxiety, social expectancy anxiety and somatic anxiety, whereas self-confidence measures an individual’s anxiety from the perspective of judgments about his or her own abilities. Thereby, leading to self-confidence was weakly correlated with IFA, SEA and SA. In general, an increase in pre-competition anxiety is not conducive to sports performance (50). Anxiety also affects an individual’s subsequent emotional state, like increased worry and negative emotions (51). This phenomenon can have a great effect on an individual’s vigor.

In addition, the indirect effect of “neuroticism→mind wandering→vigor” was significant, meaning that mind wandering mediated the relationship between neuroticism and athletes’ vigor. This result confirms hypothesis 3. In the study of Ibaceta and Madrid (39), the data collected suggests a positive correlation between neuroticism and mind wandering self-perception. In another study (35), the results showed that neurotic individuals tended to report more mind wandering during cognitive tasks. In competition, where milliseconds determine outcomes and the smallest mistake can have catastrophic consequences, the complex interplay of and physiological factors can determine the trajectory of an entire career (52). For athletes who are constantly exposed to the pressures of competition, high expectations are placed on them by their country and spectators. Psycho-social factors can reduce the ability to feel positive emotions (27), which in turn increases the frequency of mind wandering. Athletes experience mind wandering can change the final result of a competition, which is why mind wandering has received increasing attention in the field of psychology and sports. Research has shown that mind wandering episodes were related to a lower decrease in negative affect during the attentional task (37). The “Decoupling Hypothesis” of mind wandering suggests that mind wandering is decoupled from the current task and coupled to thoughts within oneself (28, 53, 54), and that if athletes focus too much on their own internal thinking or consider the event before the competition, the attention to sports task is relatively lost, and then the level of vigor devoted to sports training and competition is reduced.

The results also showed that athletes’ pre-competition anxiety and mind wandering were positively correlated, which is consistent with hypothesis 4. This finding is the same as the results of previous studies (55), which showed that anxiety is positively correlated with the frequency of mind wandering. The relationship between anxiety and mind wandering was further elaborated in a follow-up study by Figueiredo and Mattos (56), who explained that high anxiety triggers cognitive distortions, which can lead to increased worrying and rumination, and this effect can lead to an increase in the frequency of mind wandering.

Finally, pre-competition anxiety and mind wandering acted as serial mediators between neuroticism and athlete’s vigor, consistent with hypothesis 5. Neuroticism not only directly affects an individual’s vigor but also correlates with athletes’ pre-competition anxiety. Meanwhile, high pre-competition anxiety is correlated with increased mind wandering, and high mind wandering can negatively predict an individual’s vigor. According to the “Attentional Control Theory” (33), anxiety impairs an individual’s attentional control by interfering with inhibitory and switching functions, thus decreasing processing efficacy. Meanwhile, mind wandering is precisely the result of diminished attentional control leading to a shift in attention from the outside world to the inside of the individual. A subsequent theory of attentional control in sports was proposed specifically for this field. It agrees with the ideas of “Attentional Control Theory” by recognizing that anxiety interferes with attentional control. Neuroticism is sensitive to negative emotions, so athletes with neuroticism usually have a high level of anxiety and are in a highly tense state during a competition. The occurrence of mind wandering caused by anxiety can cause failure in attention control, thereby affecting athletes’ vigor level. Thus, this state of high anxiety and high mind wandering is not conducive to the vigor level of athletes with neuroticism. Taken together, the results support the hypothesis of the overall model constructed, that is, pre-competition anxiety and mind wandering act as serial mediators between neuroticism and athletes’ vigor.

The results of this study suggest that neuroticism, anxiety, and mind wandering play an important role in influencing athletes’ vigor. In sports, attention must be paid to the negative effect of this serial path on athletes. The following recommendations are made for competitive sports. First, for athletes with neurotic personalities, the anxiety level and the frequency of mind wandering could be reduced to improve the vigor level of athletes. For example, relaxation and mindfulness trainings indirectly increase vigor levels in athletes by reducing anxiety and the frequency of mind wandering. Second, the occurrence of mind wandering could be reduced by reducing the level of pre-competition anxiety. For some sporting events that require high quality of attention, mind wandering is a disruptive factor to the normal level of performance of athletes. Finally, the coaches should pay special attention to the athletes with high levels of neuroticism. They may be more likely to experience high anxiety and increase the possibility of mind wandering in the competition, eventually having a negative influence on vigor.

## Limitations and future directions

This study also has some limitations that need to be improved in the future. First, the variables in this study were measured by questionnaire. Although there are no serious common methodological biases by statistical tests, it is still possible to be affected by them. Future research needs to use a combination of experimental tasks and questionnaires to further validate the results of this study. Second, this study considered both training and competition situations when measuring mind wandering. Future studies could still accurately distinguish the situations in which mind wandering occurs. The occurrence of mind wandering in athletes during competition can have a serious negative impact on sporting performance. Future research on mind wandering will be of great value in guiding sports practice. For example, exploring the frequency, content, and other characteristics of mind wandering in competition situations could contribute to the improvement of athletes’ sports performance. Moreover, future intervention-type studies affecting athletes’ vigor could be conducted. The findings suggest that the role of emotional (anxiety) and cognitive factors (mind wandering) should be given equal weight when implementing interventions. That is, both measures should be taken to alleviate pre-competition anxiety and attention should be paid to reducing the frequency of mind wandering.

## Conclusion

In this study, the structural equation model was used to investigate the influence of neuroticism on athletes’ vigor and the role of pre-competition anxiety and mind wandering between them. We found that neuroticism had a negative effect on athletes’ vigor. Pre-competition anxiety and mind wandering mediated between neuroticism and vigor, respectively. Pre-competition anxiety was positively related to mind wandering. Pre-competition anxiety and mind wandering played serial mediation roles between neuroticism and vigor. The mechanism of neuroticism affecting athletes’ vigor was systematically analyzed, and ideas for ensuring athletes’ vigor and improving sports performance were provided.

## Data Availability

The original contributions presented in the study are included in the article/supplementary material, further inquiries can be directed to the corresponding author.

## Footnotes

^1^https://www.wjx.cn
